# Predictive factors of mortality in open abdomen for abdominal sepsis: a retrospective cohort study on 113 patients

**DOI:** 10.1007/s13304-021-01012-8

**Published:** 2021-03-08

**Authors:** Dario Tartaglia, Jacopo Nicolò Marin, Alice Maria Nicoli, Andrea De Palma, Martina Picchi, Serena Musetti, Camilla Cremonini, Stefano Salvadori, Federico Coccolini, Massimo Chiarugi

**Affiliations:** 1 Emergency Surgery Department and Trauma Center, University of Pisa, New Santa Chiara Hospital, Via Paradisa 2, 56124 Pisa, Italy; 2 Consiglio Nazionale delle Ricerche Area della Ricerca di Pisa, Pisa, Toscana Italy

**Keywords:** Open abdomen, Abdominal sepsis, Mortality, Negative pressure, Morbidity, Damage control

## Abstract

Over the past few years, the open abdomen (OA) as a part of Damage Control Surgery (DCS) has been introduced as a surgical strategy with the intent to reduce the mortality of patients with severe abdominal sepsis. Aims of our study were to analyze the OA effects on patients with abdominal sepsis and identify predictive factors of mortality. Patients admitted to our institution with abdominal sepsis requiring OA from 2010 to 2019 were retrospectively analyzed. Primary outcomes were mortality, morbidity and definitive fascial closure (DFC). Comparison between groups was made via univariate and multivariate analyses. On 1474 patients operated for abdominal sepsis, 113 (7.6%) underwent OA. Male gender accounted for 52.2% of cases. Mean age was 68.1 ± 14.3 years. ASA score was > 2 in 87.9%. Mean BMI, APACHE II score and Mannheim Peritonitis Index were 26.4 ± 4.9, 15.3 ± 6.3, and 22.6 ± 7.3, respectively. A negative pressure wound system technique was used in 47% of the cases. Overall, mortality was 43.4%, morbidity 76.6%, and DFC rate was 97.8%. Entero-atmospheric fistula rate was 2.2%. At multivariate analysis, APACHE II score (OR 1.18; 95% CI 1.05–1.32; *p* = 0.005), Frailty Clinical Scale (OR 4.66; 95% CI 3.19–6.12; *p* < 0.0001) and ASA grade IV (OR 7.86; 95% CI 2.18–28.27; *p* = 0.002) were significantly associated with mortality. OA seems to be a safe and reliable treatment for critically ill patients with severe abdominal sepsis. Nonetheless, in these patients, co-morbidity and organ failure remain the major obstacles to a better prognosis.

## Background

A wide range of pathological conditions could be related to abdominal sepsis such as generalized primary or secondary peritonitis, massive intestinal infarction and severe acute pancreatitis complicated by infected necrosis. In the context of damage control surgery (DCS), open abdomen (OA) is indicated in case of septic shock, inability to control the source of infection, the need for a deferred intestinal anastomosis, loss of abdominal wall and important visceral edema leading to abdominal compartment syndrome (ACS) [1]*.* There is no certainty on whether and when to choose OA instead of primary closure at first laparotomy with on-demand relaparotomy, even though the use of OA is increasing worldwide [2]. OA could potentially be associated with several critical complications, that can lead to a high rate of mortality in such very frail category of patients. However, the improvement of different types of temporary abdominal closure technique and a better comprehension of the pathophysiology of the OA have led to a dramatic reduction of specific complications like entero-atmospheric fistulas [3]*.* Only few observational, small cohort and non-comparative studies focused on the OA in septic patients without chasing significant conclusions. Moreover, little is in the literature focusing on the research of predictive parameters of mortality which might help surgeons to adequately select patients and choose between different therapeutic strategies. The present study aimed to evaluate postoperative outcomes of patients with abdominal sepsis treated with OA mainly in terms of perioperative mortality, overall morbidity and definitive fascial closure rates and to identify potential predictive factors of perioperative mortality.

## Methods

### Patients and setting

All patients undergoing OA with a diagnosis of abdominal sepsis (i.e., secondary or tertiary generalized peritonitis due to intestinal perforation, intestinal infarction, necrotizing infected acute severe pancreatitis, multiple abdominal abscesses) and/or septic shock in a single academic center from 2010 to 2019 were reviewed. The institutional review board approved the study design. This research complied with Ethical Standards and informed consent was obtained in all patients. Septic shock was defined according to the 3rd International Consensus Definitions for Sepsis and Septic Shock [4]. OA was adopted in case of septic shock due to abdominal peritonitis. In case of absence of shock, the decision to perform OA was taken according to the presence of massive grade of peritoneal contamination, patients’ severe comorbidities and fast deterioration of clinical conditions, which might not have allowed patient to sustain a prolonged operative duration.

### Surgical technique

Temporary abdominal closure (TAC) techniques used in this study included Negative Pressure Wound Therapy with commercial kits (NPWT), Vacuum-pack technique as described by Barker et al. [5], and Skin-closure technique. A mesh mediated NPWT was accomplished from the second revision with persistence of OA indication, or at the index laparotomy in case of patients who already had recent several surgical operations. In that case, after the intra-abdominal fenestrated plastic visceral protective layer was set in place, a 30 × 30 cm polypropylene mesh was sutured to fascial edges with a running nonabsorbable monofilament suture before setting perforated foam and adhesive drapes. At the following look, the mesh was divided along the main axis in two halves, the visceral drape pulled out, and the peritoneal cavity carefully evaluated. In case of definitive closure, the mesh was removed after removing the running nonabsorbable suture in the fascial edges on each side; otherwise, the two halves of the mesh were stretched to bring near the fascial edges as much as possible and then joined with a median running nonabsorbable suture [6]. The decision to close the wall or to continue OA at the second look was left to the attending surgeon, on the basis of an exhaustive control of the infectious source, patient’s substantial clinical improvement, and vitality of the abdominal organs. Successful definitive fascial closure was defined as complete closure of the whole length of the incised fascia.

### Patient data and follow-up

**Fig. 1:**
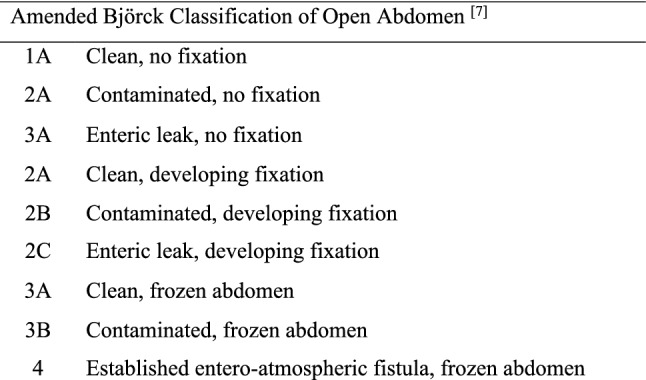
Amended Björck classification of open abdomen [7]

Data collected for each patient included gender, age, Body Mass Index, comorbidities, clinical and prognostic scores such as American Society of Anesthesiologists (ASA) score, Charlson Age-Comorbidity (CaCI) Index, Acute Physiology And Chronic Health Evaluation II (APACHE II), time to surgery (in hours), Frailty Clinical Scale, and Mannheim Peritonitis Index (MPI). OA indications, TAC techniques, number of looks, modified Björck classification at second look (Fig. 1) [7], definitive fascial closure and cutaneous closure, use of a prosthetic mesh, in-hospital overall morbidity, ICU length of stay (LOT), 30-day reintervention rate, and in-hospital mortality were also collected and analyzed. All patients included in this study were followed up for 1 year from hospital discharge. Follow-up evaluation included outpatient clinic visits and/or phone interviews.

### Statistical analysis

Categorical variables were presented as numbers and percentages, whereas continuous variables as mean ± standard deviation if uniformly distributed, and median and interquartile range (IQR) if not uniformly distributed. Association analysis between mortality and variables potentially affecting outcome (gender, age, BMI, CaCI index, comorbidities, ASA score, MPI, APACHE II, time to surgery, Frailty Clinical Scale, TAC technique, modified Björck grade at second look, number of surgical looks, OA duration, ICU LOS) was carried out. Continuous variables were compared with Student *t* test for independent variables, both homoscedastic and heteroscedastic version, and Mann–Whitney *U* test, as appropriate. Normality of distributions were assessed through Shapiro–Wilk test. Categorical variables were compared by Chi-squared test. Comparison between mortality during OA and after DFC was carried out with a two-sample proportion *z* test. A multivariate analysis was carried out with a binary logistic regression model in stepwise backward mode. Significance level was set at *p* < 0.05 both at univariate and multivariate analysis. All statistics were processed by SPSS software 24.0 version (IBM^®^).

## Results

On 1474 patients operated for severe abdominal sepsis, 113 patients (7.6%) were analyzed. Fifty-nine patients (52.2%) were male. Mean age was 68.1 ± 14.3 years and mean BMI resulted 26.4 ± 4.9 kg/m^2^ (Table 1). Comorbidities were present in 96.1% of patients and were mainly represented by arterial hypertension in 38.2% of cases, cancer in 29.4%, cardiac disease in 20.6%, pulmonary disease in 17.6%, diabetes mellitus in 10.7% and immune disorders in 10.8%. Mean CaCI score was 4.5 ± 2.3. An ASA score = IV was reported in 50.5% of cases. Mean MPI was 22.6 ± 7.3 and mean APACHE II was 15.3 ± 6.3. Mean time to surgery was 9.2 ± 6.7 h. Median Frailty Clinical Scale was 7 (IQR 1–9).

**Table 1:** Population characteristics

Patients	*N* = *113*
Male gender, *n* (%)	59 (52.2)
Age, mean ± SD	68.1 ± 14.3
BMI, mean ± SD	26.4 ± 4.9
Charlson age—comorbidity index, mean ± SD	4.5 ± 2.3
Comorbidities, *n* (%)	
Hypertension	39 (38.2)
Cancer and/or chemotherapy	30 (29.4)
Cardiopathy/cardiomyopathy	21 (20.6)
Diabetes	15 (14.7)
Pneumological disorders	18 (17.6)
Obesity	12 (11.8)
Immunological disorders	11 (10.8)
Neurological disorders	10 (9.8)
Hepatopathy	8 (7.8)
Nephropathy	7 (6.9)
Smoking	4 (3.9)
Malnutrition	3 (2.9)
Immunosuppression/steroid use	3 (2.9)
Aneurism	3 (2.9)
Presence of ostomy	4 (3.9)
None	4 (3.9)
Other	46 (45.1)
ASA, *n* (%)^a^	
I	1 (1.0)
II	11 (11.1)
III	37 (37.4)
IV	50 (50.5)
Mannheim peritonitis index, mean ± SD	22.6 ± 7.3
APACHE II score, mean ± SD	15.3 ± 6.3
Time to surgery (hours), mean ± SD	9.2 ± 6.7
Frailty clinical scale, median (IQR)	7 (1–9)

*BMI* body mass index, *ASA* American society of Anesthesiology score, *APACHE II* acute physiology and chronic health evaluation II score, *SD* standard deviation, *IQR* interquartile range

^a^Based on 99 pts with available ASA score data

Seventy-one (62.8%) patients presented with a bowel perforation, 29 (25.7%) bowel infarction, 10 (8.8%) multiple abdominal abscesses and 3 (2.7%) an infected necrotizing acute severe pancreatitis.

**Fig. 2:**
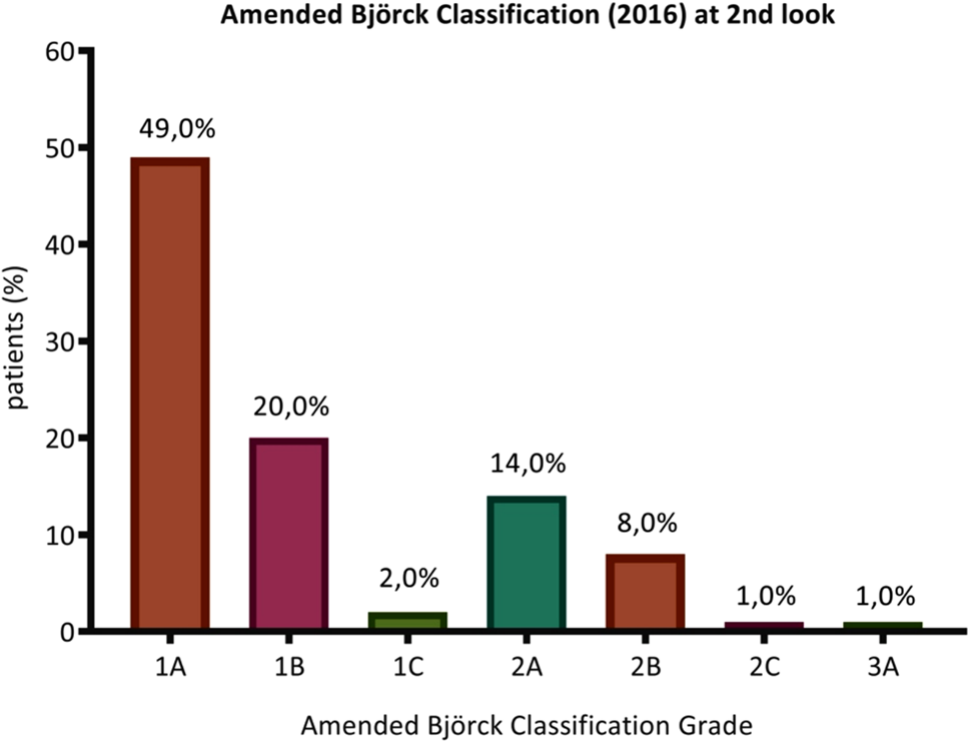
Distribution of amended Björck classification grades at 2nd look

In this study, 46.9% of cases were treated with NPWT technique, 33.6% with Vacuum-pack technique, 15.9% with skin-closure, and 3.5% with mesh-mediated NPWT since the first look (Table 2). Mean OA duration was 2.8 ± 1.7 days, with a mean number of looks of 1.2 ± 0.8. The distribution of the modified Björck classification at the 2^nd^ look is described in the Fig. 2: 49% of cases presented a 1A grade.

**Table 2:** Perioperative variables during OA treatment

TAC technique adopted at first look, *n* (%)	
NPWT with commercial kits	53 (46.9)
Vacuum-pack technique	38 (33.6)
Skin-closure	18 (15.9)
Mesh-mediated NPWT	4 (3.5)
OA* duration, mean ± SD	2.8 ± 1.7
Number of looks, mean ± SD	1.2 ± 0.8
ICU length of stay, mean ± SD	13.7 ± 12.8
Type of nutrition, *n* (%)	
Parenteral	96 (95.0)
Enteral	2 (2.0)
Enteral + parenteral	3 (3.0)

*TAC* temporary abdominal closure, *NPWT* negative pressure wound technique, *OA* open abdomen, *ICU* intensive care unit, *SD* standard deviation

Morbidity occurred in 76.6% of cases. The overall mortality was 43.4% (Table 3). Twenty patients (40.8%) deceased during OA treatment, while 29 (59.2%) deceased during the first 30 days from the definitive fascial closure (*p* = 0.02). The causes of death were cardiopulmonary complications (55%), multiorgan failure due to sepsis (41%), and irreversible brain damage (4%) (Table 3). There was no mortality between 30 and 90 days. Among OA surviving patients, 97.8% of cases reached definitive fascial closure. Ten patients (10.8%) required a prosthetic mesh (absorbable polyglactin 910 mesh: suprafascial in 5 and intraperitoneal in 3 cases; polypropylene retromuscular mesh in 1 case, and bioabsorbable intraperitoneal mesh together with polypropylene suprafascial mesh in 1 case). In 94.6% of cases, the skin was contextually closed.

**Table 3:** Patients’ outcomes

Perioperative mortality, *n* (%)	
Overall	49/113 (43.4)
During OA	20/49 (40.8)
After definitive closure	29/49 (59.2)
Causes of mortality, *n* (%)	
Cardiopulmonary complications	
Overall	27/49 (55)
During OA	8/20 (40)
After definitive closure	19/29 (65)
Multiorgan failure due to sepsis	
Overall	20/49 (41)
During OA	11/20 (55)
After definitive closure	9/29 (31)
Irreversible brain damage	
Overall	2/49 (4)
During OA	1/20 (5)
After definitive closure	1/29 (4)
Definitive fascial closure, *n* (%)	91/93 (97.8)
Definitive cutaneous closure, *n* (%)	88/93 (94.6)
Prosthetic mesh, *n* (%)	10/93 (10.8)
Overall postoperative complications, *n* (%)	49/64 (76.6)
Reinterventions, *n* (%)	20/93 (21.5)
Entero-atmospheric fistula, *n* (%)	2/93 (2.2)

*OA* open abdomen

After definitive fascial closure, 30-day reintervention rate was 21.5%. The reasons were represented by wound dehiscence (5 cases), tertiary peritonitis (7 cases), hemorrhage (5 cases), multiple abdominal abscesses (1 case), bowel ischemia (1 case) and urinary fistula (1 case). Two patients (2.2%) developed an entero-atmospheric fistula. Mean ICU LOS was 13.7 ± 12.8 days.

According to the time to surgery, there was no significant difference in patients who died (9.63 ± 7.16 h) and those who survived (8.87 ± 6.48 h) (*p* = 0.712) The same was for patients who experienced a complication (9.48 ± 7.02 h) vs. who did not (6.71 ± 3.38 h) (*p* = 0.237). Frailty Clinical Scale was 4 (IQR 2–9) in non-survived and 3 (IQR 1–8) in survived (*p* = 0.001). Conversely, no significant differences were found between complicated vs. non-complicated patients: 3 (IQR 1–8) vs. 3 (IQR 2–7) (*p* = 0.632). At the univariate analysis, other factors influencing mortality were age (*p* = 0.05), neurological disorders (*p* = 0.05), ASA IV (*p* = 0.04) and APACHE II score (*p* = 0.001) (Table 4).

**Table 4:** Univariate analysis between survivors and non-survivors groups

	Survivors *N* = 64	Non-survivors *N* = 49	*p*
Male gender, *n* (%)	33 (51.6)	26 (53.1)	0.87
Age, mean ± SD	65.8 ± 14.0	71.2 ± 14.3	*0.05*
BMI, mean ± SD	26.6 ± 5.0	26.1 ± 4.6	0.59
Charlson age-comorbidity, mean ± SD	4.2 ± 2.6	5.0 ± 1.8	0.07
Comorbidities, *n* (%)			
Hypertension	23 (37.1)	16 (40.0)	0.77
Cancer and/or chemotherapy	20 (32.3)	10 (25.0)	0.43
Cardiopathy/cardiomyopathy	10 (16.1)	11 (27.5)	0.17
Diabetes	9 (14.5)	6 (15.0)	0.95
Pneumological disorders	9 (14.5)	9 (22.5)	0.30
Obesity	8 (12.9)	4 (10.0)	0.76
Immunological disorders	7 (11.3)	4 (10.0)	1.00
Neurological disorders	3 (4.8)	7 (17.5)	*0.05*
Hepatopathy	3 (4.8)	5 (12.5)	0.16
Nephropathy	3 (4.8)	4 (10.0)	0.43
Smoking	4 (6.5)	0 (0)	0.15
Malnutrition	1 (1.6)	2 (5.0)	0.56
Immunosuppression/steroids	0 (0)	3 (7.5)	0.06
Aneurism	0 (0)	3 (7.5)	0.06
Presence of ostomy	4 (6.5)	0 (0)	0.15
Other	29 (46.8)	17 (42.5)	0.67
None	4 (6.5)	0 (0)	0.15
ASA IV^a^, *n* (%)	23 (35.9)	27 (55.1)	***0.04***
Mannheim Peritonitis Index ≥ 30^b^, *n* (%)	6 (9.4)	9 (18.4)	0.16
APACHE II score, mean ± SD	13.2 ± 5.3	18.4 ± 6.6	***0.001***
Time to surgery (hours), mean ± SD	8.87 ± 6.48	9.63 ± 7.16	0.71
Frailty clinical scale, median (IQR)	3 (1–8)	4 (2–9)	***0.001***
TAC technique, *n* (%)			0.29
NPWT	27 (42.2)	26 (53.1)	
Vacuum-pack technique	24 (37.5)	14 (28.6)	
Skin-closure	12 (18.8)	6 (12.2)	
Mesh-mediated NPWT	1 (1.6)	3 (6.1)	
1A Björck’s grade at 2nd look^c^, *n* (%)	31 (48.4)	18 (36.7)	0.21
Number of looks, mean ± SD	1.3 ± 0.6	1.1 ± 1.1	0.15
OA duration, mean ± SD	2.9 ± 1.8	2.6 ± 1.6	0.54
ICU length of stay, mean ± SD	14.5 ± 10.6	12.4 ± 15.5	0.44

In **bold** are reported *p *values, rounded to second decimal, inferior to significance level

*BMI* body mass index, *ASA* American society of anesthesiology score, *APACHE II* acute physiology and chronic health evaluation II score, *TAC* temporary abdominal closure, *NPWT* negative pressure wound technique, *OA* open abdomen, *ICU* intensive care unit, *SD* standard deviation, *IQR* interquartile range

^a^The analysis was carried out by two levels “IV” and “III or less”, considering the distribution of the four levels within the sample

^b^The analysis was carried out by two levels “30 or over” and “29 or less”, considering the distribution within the sample

^c^The analysis was carried out by two levels “1A” and “1B or over”, considering the distribution within the sample

At the multivariable logistic regression analysis, an increase of 1 point of APACHE II score was associated with an increase of OR of perioperative mortality by 1.18 (95% CI 1.05–1.32; *p* < 0.05). Frailty Clinical Scale (OR 4.66; 95% CI 3.19–6.12; *p* < 0.0001) and ASA IV (OR 7.86; 95% CI 2.18–28.27; *p* < 0.05) were also associated with an increased risk of perioperative mortality (Table 5).

**Table 5:** Multivariate analysis

	OR	95% CI	*p*
Step 1			
Age	0.99	0.94 – 1.04	0.73
Male	1.29	0.32 – 5.13	0.72
Neurological disorders	2.26	0.27 – 19.23	0.46
MPI	2.63	0.51 – 13.53	0.25
Frialty clinical scale	0.60	0.12 – 1.08	*0.013*
Apache II	1.20	1.05 – 1.37	*0.008*
ASA IV	6.74	1.78 – 25.47	*0.005*
Step 5			
Frialty clinical scale	4.66	3.19 – 6.12	< *0.0001*
Apache II	1.18	1.05 – 1.32	*0.005*
ASA			
IV	7.86	2.18 – 28.27	*0.002*
III or less	1.00		

Analysis is carried out with a binary logistic regression model with *Stepwise backward* selection mode. In **bold** are reported *p* values inferior to significance level

*MPI* Mannheim peritonitis index, *ASA* American society of anesthesiology score, *APACHE II* acute physiology and chronic health evaluation II s

At 1-year follow-up, 23/64 patients (36%) died neither for surgical reasons nor for other causes related to the open abdomen.

## Discussion

This study shows that OA in septic patients is feasible and allows a high rate of fascial closure, despite high morbidity and mortality. APACHE II, Frailty Clinical Scale, and ASA score IV could be considered as predictive factors for mortality in patients undergoing OA for septic shock due to abdominal diseases.

The overall cohort of patients presented in this study represents a very frail and complex population. Firstly, patients presented a distribution largely shifted to more advanced age groups. Consequently, the presence of multiple comorbidities was a common finding. They were mostly represented by cardiovascular, pulmonary and neoplastic diseases and were more frequent than in other series reported [2, 8, 9]. Secondly, mean BMI was high and a 9% of patients presented a severe obesity. Thirdly, an ASA score greater than II was recognized in the majority of patients (88.3%).

According to the most recent guidelines, primary abdominal closure must be performed within the first 8 days of treatment [10]. In our series, only 25% of patients required more than two surgical revisions and definitive fascial closure was obtained within 3 days in 97.8% of patients. The definitive closure rate shown in our series is higher than the one reported in the literature [11–14]. In their review, Atema et al. reported that the delayed fascial closure rate was described in 63 of the 78 included series and ranged from 3.2 to 100% with an overall weighted closure rate of 50.2%. This finding could be related to a poor overall quality and a substantial heterogeneity of the included studies [13], along with a possible bias in the methodology of some of the considered studies, that included patients who died during OA inside the cohort of patient in which the fascia was not closed. The use of negative pressure systems, characterized by a greater efficacy in terms of definitive fascial closure [11, 13, 14], and the progressive experience gained in the management of OA were also crucial elements positively influencing the 97.8% of DFC rate in our series.

The high overall complication rate (76.6%) found in our series represents one of the critical factors that must be considered in septic patients treated with OA. Coccolini et al. showed, on 402 prospectively collected patients, a complication rate of 38% during OA and 49.5% after closure [9]. However, the international register of open abdomen included patients with different etiologies: peritonitis (48.7%), trauma (20.5%), vascular emergencies/hemorrhage (9.4%), ischemia (9.1%), pancreatitis (4.2%), post-operative abdominal-compartment-syndrome (3.9%), and others (4.2%). Furthermore, several temporary-abdominal-closure systems were taken into account and were mainly represented by the commercial negative pressure ones (44.2%).

Entero-atmospheric fistulas represent a dramatic complication of OA, as mortality in such situation is reported to be as high as 42% [15]. In our series, the entero-atmospheric fistula rate (2.2%) was notably lower than previously published in the literature (up to 26%) [9, 11, 14, 16]. Giudicelli et al. reported on 57 patients with different types of disease undergoing OA with NPWT, a 14% of EAF rate and a median duration of laparostomy of 12 days. They identified the presence of mesenteric ischemia as a potential risk factor for EAF formation [16]. Even though the natural history and predictors of EAF formation in the OA are largely unknown, we could speculate that our low EAF rate may be related to a relatively short OA duration (2.8 days).

The mortality rate observed in our series is consistent with previously published literature [9]. However, we observed a lower mortality rate during the condition of open abdomen rather than after definitive fascial closure (40.8% vs. 59.2%; *p* = 0.02). This finding could be explained by the complicated postoperative management of these patients, which is further limited by the lack of evidence currently available [17–19]. Indeed, a further evidence that mortality was not only a direct, immediate, consequence of the ongoing abdominal sepsis and the treatment adopted, was the fact that no differences in terms of OA duration, number of looks and Björck classification were found between survivors and non-survivors. Another explanation may be the difficult selection of patients who can benefit from treatment with OA because of the insufficiency of reliable prognostic scores and unique clinical indications [20].

The high average values of ASA score, APACHE II and MPI confirm the physiological impairment of patients included in this study, as reported in other studies [8, 21]. Univariate and multivariate analysis revealed three variables independently associated with mortality: APACHE II score (OR 1.18; 95% CI 1.05–1.32; *p* = 0.005), Frailty Clinical Scale (OR 4.66; 95% CI 3.19–6.12; *p* < 0.0001) and an ASA score IV (OR 7.86; 95% CI 2.18–28.27; *p* = 0.002). These findings confirm the utility of these scores in critical patients’ management. As matter of facts, a critical physiological impairment should be carefully taken into account in the decision to perform or not an OA, as mortality appears not to significantly decrease in this extremely weakened category of patients. In a recent study on 101 septic patients treated with OA by Morais et al., APACHE II score and age older than 60 years resulted as strong predictive factors of mortality at the multivariate analysis. Furthermore, they found that greater number of reinterventions and longer ICU stay were associated with inability to primarily close the fascia. As a consequence, the authors concluded that the recognition of these risk factors should be promoted to guarantee a tailored surgical approach in these patients [22]. In addition, Tolonen et al. identified as significantly factors associated to mortality, advanced age, higher Charlson Comorbidity Index, preoperative organ dysfunctions, higher MPI, prophylactic indication for OA, and higher SOFA scores in the ICU. Moreover, the authors stated that these results aligned with previously recognized risk factors [23].

The present study has several limitations which are represented by its retrospective nature, the small sample size and the fact that it has been conducted in a single tertiary center, which may be related to centripetal bias. However, the results emerging from the present research may be useful in future reviews to better identify which patients mostly benefit from this approach and pose a base for a large-scale multi-institutional study.

## Conclusions

The open abdomen in septic patients is still an open challenge. In critically ill patients undergoing OA for severe abdominal sepsis, postoperative mortality and overall complication rates remain high, despite fascial closure could be achieved in almost all patients. APACHE II score, Frailty Clinical Scale and ASA class IV have been recognized as the only independent predictive factors of mortality. A critical physiological impairment associated with severe patient’s comorbidities should be carefully evaluated before deciding to perform an OA or not. It would be interesting in future studies, to separately analyze mortality predictors during the treatment with OA and after the final closure of the abdomen.
